# *MiR-196a2* rs11614913 C Allele is Associated with Increased Wilms Tumor Susceptibility in Chinese Children

**DOI:** 10.7150/jca.102801

**Published:** 2025-01-01

**Authors:** Ming Luo, Yizhen Wang, Huimin Yin, Chunlei Zhou, Hongting Lu, Haixia Zhou, Shaohua He, Jing He, Jinhong Zhu, Shouhua Zhang

**Affiliations:** 1 Department of General Surgery, Jiangxi Provincial Children's Hospital, Nanchang 330006, Jiangxi, China.; 2 Department of Pathology, Anhui Provincial Children's Hospital, Hefei 230051, Anhui, China.; 3 Department of Pediatric Surgery, Guangzhou Institute of Pediatrics, Guangdong Provincial Key Laboratory of Research in Structural Birth Defect Disease, Guangzhou Women and Children's Medical Center, Guangzhou Medical University, Guangzhou 510623, Guangdong, China.; 4 Department of Pathology, Children's Hospital of Nanjing Medical University, Nanjing 210008, Jiangsu, China.; 5 Department of Pediatric Surgery, The Affiliated Hospital of Qingdao University, Qingdao 266000, Shandong, China.; 6 Department of Hematology, The Key Laboratory of Pediatric Hematology and Oncology Diseases of Wenzhou, The Second Affiliated Hospital and Yuying Children's Hospital of Wenzhou Medical University, Wenzhou 325027, Zhejiang, China.; 7 Department of Pediatric Surgery, Shengli Clinical Medical College of Fujian Medical University, Fuzhou 350001, Fujian, China.; 8 Department of Clinical Laboratory, Biobank, Harbin Medical University Cancer Hospital, Harbin 150040, Heilongjiang, China.

**Keywords:** Wilms tumor, susceptibility, *miR-196a2*, rs11614913, polymorphism

## Abstract

Wilms tumor, also known as nephroblastoma, is the most common kidney cancer in children. The* miR-196a2* rs11614913 T>C polymorphism has been identified as a susceptibility locus in various adult cancers. However, it is unclear whether this polymorphism also increases the risk of pediatric cancer. In this study, we examined the genotypes of *miR-196a2* rs11614913 T>C in 416 pediatric patients with Wilms tumor and 936 age- and gender-matched healthy controls of Chinese Han ethnicity using the TaqMan technology. The association between rs11614913 T>C polymorphism and the susceptibility to Wilms tumor was analyzed by univariable and multivariable logistic regression analyses, and the odds ratio (OR) and 95% confidence interval (CI) were calculated. Overall analysis indicated that the *miR-196a2* rs11614913 T>C was associated with an increased risk of Wilms tumor in the homozygous (adjusted OR (AOR)=1.58, 95% CI=1.14-2.21, *P*=0.007), additive (AOR=1.26, 95% CI=1.06-1.49, *P*=0.007), dominant (AOR=1.33, 95% CI=1.02-1.74, *P*=0.034), and recessive (AOR=1.38, 95% CI=1.05-1.81, *P*=0.023) models. Stratification analysis further demonstrated that the association was dependent on age and tumor stages rather than gender. Furthermore, *miR-196a2* rs11614913 T>C was significantly associated with the disease risk only in children older than 18 months and those with III+IV stage tumors. The expression quantitative trait locus (eQTL) analysis showed that rs11614913 T>C was associated with decreased expression of HOXC-AS1 and increased expression of GPR84 and HOXC8. Our results provide evidence that *miR-196a2* rs11614913 T>C may confer genetic susceptibility to Wilms tumor. These findings warrant validation in larger cohorts and across different ethnicities.

## Introduction

Wilms tumor, also referred to as nephroblastoma, is a pediatric kidney cancer stemming from the immature cells in the developing kidney. The age-standardized incidence rates (ASR) of Wilms tumor have been varying from 9.1 to 9.8 cases per million in North America and Europe and around 4.3 in East Asia annually 1, 2. It is the most frequently diagnosed childhood kidney cancer 3. The prognosis for Wilms tumor is generally optimistic as a result of early diagnosis and multimodality regimes, including surgery, radiation, and chemotherapy 4. However, relapse, late effects of treatment, as well as a subgroup of high-risk patients with a survival rate of 50% or lower remain essential long-term concerns. Current research focuses on improving the management of relapsed or refractory tumors, as well as minimizing the adverse effects of therapy in survivors.

Genetic risk factors for Wilms tumor development have been reported, such as syndromes and genetic abnormalities. Common predisposing syndromes include WAGR, Denys-Drash, and Beckwith-Wiedemann syndromes 3. Moreover, mutations of *WT1*, *CTNNB1*, *ASXL1*, *MAP3K4*, and *WTX* genes and loss of *H19-IGF2* imprinting can help explain a subset of patients 5. Besides, many Wilms tumor-predisposing loci have also been identified, such as single nucleotide polymorphisms (SNPs) in the *RAN, RANBP2*, *miR34b/c*, *TP53*, *TRMT6*, *YTHDF2*, and *WTAP* genes 6-14. However, more studies are warranted to understand pediatric renal tumors better, dissect more risk factors, and develop more risk-adapted therapies.

Apart from SNPs of protein-encoding genes, genetic variants in non-coding RNA genes are also nonnegligible. MicroRNAs, evolutionarily conserved small non-coding RNAs, impede the translation or stability of mRNAs by binding to the target transcript's 3' untranslated region (UTR) and then negatively regulate gene expression. MicroRNAs approximately regulate more than 30% of gene expression whose abnormal expression is tightly related to various diseases, including cancer 15, 16. A genome-wide suppression of miRNA maturation leads to cellular transformation and carcinogenesis 17. miRNAs are involved in the pathogenesis of Wilms tumor 18. Alterations in miRNA expression profiles have been observed in Wilms tumor tissues compared to normal kidney tissues, suggesting that miRNAs play a significant role in the tumor's biology 19, 20. *miR-378c*, *miR-204*, and *miR-483-5p* are among the miRNAs previously reported to be dysregulated in Wilms tumor 21, 22. These miRNAs are involved in key pathways that promote tumorigenesis, such as cell cycle regulation, apoptosis evasion, and angiogenesis, underscoring the critical role of miRNA dysregulation in the disease 20, 23, 24. SNPs within miRNAs (e.g., seed region) or nearby microRNA binding sites of functional genes can compromise or strengthen a microRNA function, thereby interfering with the miRNA-targeted mRNAs, such as altered expression of tumor suppressors, oncoproteins, or genes-related to drug sensitivity. Moreover, SNPs in pri- and pre-microRNAs may also modulate the processing and maturation of microRNAs 25, 26. These variations can modulate individual susceptibility to various cancers. Studies have identified several miR-SNPs associated with altered cancer risk, such as *miR-499* rs3746444, *miR-146a* rs2910164, and *miR-423* rs6505162 11, 27, 28. The association of *miR-196a2* SNPs with disease risk has been investigated in a broad of cancers, including breast cancer 29, esophageal carcinoma 30, non-small cell lung cancer (NSCLC) 31, glioma 32, colorectal cancer (CRC) 33. However, its roles in pediatric solid cancer are much less investigated 34.

This study aimed to interrogate whether the *miR-196a2* rs11614913 T>C polymorphism is associated with Wilms tumor susceptibility in 416 cases and 936 matched healthy controls. The impacts of the rs11614913 polymorphism on the expression of neighboring genes were also investigated using expression quantitative traits locus (eQTL) analysis.

## Methods and Materials

### Study population

This Chinese Han children study cohort consisted of 416 cases and 936 controls. To meet inclusion criteria, cases were diagnosed with Wilms tumor based on histopathological confirmation and had not received any prior chemotherapy, radiotherapy, or targeted therapy before sample collection. Cases were excluded if there was a simultaneous presence of other malignancies or significant comorbid conditions. Healthy controls should be free of any history of malignancies or other chronic diseases, as well as genetic conditions associated with increased Wilms tumor risk. Children with abnormal clinical examination findings or laboratory tests suggesting underlying diseases were excluded. Cases meeting the inclusion criteria were recruited from various medical centers in East China, situated in Anhui, Jiangsu, Zhejiang, Shandong, Fujian, and Jiangxi provinces. Accordingly, 936 age- and gender-matched healthy controls were enrolled from the participating hospitals during the same periods. We conducted this study in accordance with the Declaration of Helsinki. The study protocol was reviewed and proved by the Ethics Committee of Guangzhou Women and Children Medical Center (No. 202016601). Parents or equally legal guardians of all participants were informed and signed the written consent. Venous blood was then withdrawn from each subject prior to any medical intervention.

### SNP selection and genotyping

We chose the *miR-196a2* SNP rs11614913 (T>C) for this study based on a literature review 26, 29-35. We first obtain genomic DNA from the above-mentioned blood samples using the Genomic DNA kit (Tian Gen Biotech Co. Ltd., Beijing, China). Then, we used a spectrophotometer to measure the DNA concentration of each sample, and then adjusted it to match the desired concentration level. The SNP of interest was genotyped with TaqMan real-time polymerase chain reaction (PCR) in 384-well plates, as reported in published studies 36-38.

### Statistical analysis

A chi-square goodness-of-fit test was used to check whether the genotype frequency distribution of the *miR-196a2* rs11614913 (T>C) polymorphism is consistent with Hardy-Weinberg equilibrium. Logistic regression was employed to estimate the strength of the association between the SNP and Wilms tumor susceptibility. The odds ratio (OR) and 95% confidence interval (CI) of the associations were calculated under different genetic models, including heterozygous, homozygous, additive, dominant, and recessive models. All models used age, gender, and clinical stage as covariates. Statistical analyses were executed utilizing SAS v10.0 (SAS Institute Inc., Cary, NC) and we recognized results with a *P*-value of less than 0.05 (two-sided) as statistically significant.

### Expression quantitative traits locus (eQTL) analysis

An eQTL is considered to be a genetic variant (a locus) or region that influences the level of activity of a particular gene. The Genotype-Tissue Expression (GTEx) project contains a comprehensive database of eQTLs for various types of tissues and cell types 39, 40. In this study, we identified genes that could be potentially affected by the *miR-196a2* SNP rs11614913 T>C, using the GTEx Portal (https://gtexportal.org/home/).

## Results

### Participant characteristics

The baseline characteristics of all subjects are provided in **Table S1**. The median age of cases and controls were 34.09±26.35 and 33.87±30.88 months, respectively. In summary, 29.81% of patients had stage I disease, 34.86% stage II, 18.51% stage III, and 9.13% had stage IV. Tumor stage information was not available for 7.69% of patients. The distribution frequencies of age, gender, and tumor clinical stages are displayed in **Table S1**.

### Association study

Different types of genetic inheritance patterns were employed to test the association between the rs11614913 T>C polymorphism and susceptibility to Wilms tumor (**Table 1**). Overall analysis indicated that the *miR-196a2* rs11614913 T>C was associated with an increased risk of Wilms tumor under homozygous (adjusted OR (AOR)=1.58, 95% CI=1.14-2.21, *P*=0.007), a model used to identify genetic factors that have a substantial effect on disease risk (**Table 1**). We also detected a significant association between the rs11614913 and the risk of Wilms tumor under dominant (AOR=1.33, 95% CI=1.02-1.74, *P*=0.034) and recessive (AOR=1.38, 95% CI=1.05-1.81, *P*=0.023) models. In addition, this SNP was also shown to elevate Wilms tumor risk under the additive (AOR=1.26, 95% CI=1.06-1.49, *P*=0.007), indicating that the effect of the rs11614913 on the disease risk increases linearly with the number of C alleles. The stratification analysis indicated that the link between Wilms tumor susceptibility and *miR-196a2* rs11614913 T>C was influenced by age and clinical stage, with no differences observed by gender (**Table 2**). A significant association was only detected in children > 18 months of age (AOR=1.45, 95% CI=1.03-2.04, *P*=0.032) and subgroup with tumors in the III+IV stage (AOR=1.92, 95% CI=1.17-3.16, *P*=0.01). These results suggest that older children with rs11614913 C allele are likely to have Wilms tumor. Moreover, children with this risk allele tended to develop advanced tumors (III+IV stage).

### Gene tissue expression analysis

**Figure 1:**
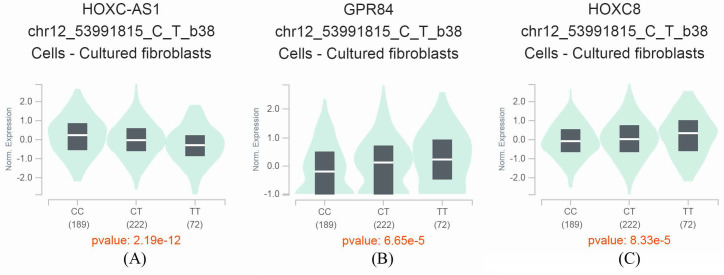
GTEx Portal analysis shows that the miR-196a2 rs11614913 T>C variant affects the expression of neighboring genes. A. HOXC cluster antisense RNA 1 (HOXC-AS1). B. inflammation-related G protein-coupled receptor EX33/G-protein coupled receptor 84 (GPR84). C. a transcription factor homeobox protein HOX-C8 (HOXC8).

By taking advantage of the GTEx Portal, we observed that the *miR-196a2* rs11614913 T>C was associated with expression levels of several neighboring genes, including HOXC cluster antisense RNA 1 (HOXC-AS1), inflammation-related G protein-coupled receptor EX33/G-protein coupled receptor 84 (GPR84), and a transcription factor homeobox protein HOX-C8 (HOXC8). With an increase in C alleles, the HOXC-AS1 expression levels decrease, and the levels of GPR84 and HOXC8 increase significantly (**Figure 1A-C**). These results suggest that the *miR-196a2* rs11614913 is an eQTL that may have impacts on the expression of these essential genes.

## Discussion

In the present study, we identified the rs11614913 T>C in the *miR-196a2* as a medium penetrant SNP in Wilms tumor, showing a moderate correlation with the risk of developing the disease. Overall analysis indicated that the rs11614913 T>C significantly conferred an increased susceptibility to Wilms tumor. The association was reliant on age and clinical stage rather than gender.

The rs11614913 polymorphism in the 3p mature miRNA region of *hsa-mir-196a2* is a functional miRNA gene polymorphism. This SNP impacts the interaction between the mature hsa-mir-196a2-3p and its target mRNA. For instance, LSP1 is a predicated target gene of *hsa-mir-196a2*. Compared to *hsa-mir-196a2* expression plasmids (T allele), the plasmids carrying rs11614913 C could significantly suppress the expression of LSP1 in Chinese hamster ovary (CHO), 293T, and A549 cell lines transfected with LSP1 3′UTR luciferase reporter plasmids, which was also validated with mature hsa-mir-196a2-3p miRNAs (C or U allele) 26. Several pieces of evidence have shown that the rs11614913 was associated with the expression of mature miR-196a2 26, 35. The study on the correlation between the rs11614913 genotype and phenotype in 23 human lung cancer tissue samples revealed that tissues with the rs11614913 CC allele showed a substantial change in mature hsa-mir-196a expression. However, no alterations were detected in the precursor levels, indicating a potentially enhanced processing of pre-miRNA into its mature format 26. Similarly, analyzing 50 CRC tissues showed that the expression level of mature miR-196a2 was significantly increased in CRC tumor tissues with rs11614913 TT compared to those with CC genotypes 35. Recently, Yueh reported a significant genotype-phenotype of serum miR-196a2 in healthy controls, with the rs11614913 CC homozygotes showing significantly higher levels of serum miR-196a2 than CT heterozygous and TT homozygous patients 33.

The frequency of the T allele of the rs11614913 shows notable variation across various ethnic groups: approximately 18.8% in Africans, 39.4% in Europeans, 41.1% in Mexicans, 30.7% in South Asians, and 54.8% in East Asians.^33^. Consistently, our controls had a T allele frequency of 58.59, close to East Asians (54.8%). The T allele represents the major allele in East Asians but is the minor allele in all other ethnicities. The clinical implications of the rs11614913 have been extensively investigated in adult cancers. The study conducted by Hu *et al.* revealed that individuals with NSCLC who have the CC homozygote genotype of rs11614913 had poorer survival outcomes than those with the combined CT heterozygote and TT homozygote genotypes 26. A systematic meta-analysis indicated that the rs11614913 (miRNA-196a2) was significantly associated with breast cancer susceptibility in Asia and South America based on data collected from Brazil, China, India, Iran, and Saudi Arabia 29. Liu *et al.* observed no association between the *miR-196a2* rs11614913 and susceptibility to esophageal squamous cell cancer (ESCC) in a study population of 829 cases and 1522 controls of Chinese Han nationality 30. However, after adjustment for age, sex, smoking, drinking status, and body mass index (BMI), this SNP seemed to be associated with reduced ESCC susceptibility in women and in the subgroups aged 63 years old or beyond 30. Yueh *et al.* found that the *miR-196a2* rs11614913 CC genotype was associated with an increased CRC risk by 2.04 folds in Taiwan 33. So far, no association between the SNP and renal cancer risk has been reported. Regarding pediatric cancer, our results in a previous study with 312 cases and 762 healthy controls suggested that the *miR-196a2* rs11614913 might not be a causal genetic variant for neuroblastoma susceptibility in Eastern Chinese children 34. Taken together, the association of rs11614913 with cancer risk may be tissue- and ethnic-specific. However, the sampling bias or other factors should be ruled out before the conclusion.

Finally, eQTL analysis demonstrated the potential association of the rs11614913 with *HOXC-AS1*, *GPR84*, and *HOXC8*. *HOXC8* is a member of the homeobox family of genes that are highly conserved across species and encode a group of transcription factors responsible for regulating morphogenesis. Four *HOX* gene clusters, *HOXA*, *HOXB*, *HOXC,* and *HOXD*, have been characterized in mammals. The gene clusters are positioned on distinct chromosomes and comprise a series of 9 to 11 genes aligned consecutively. Interestingly, miR-196 was reported to be encoded at three paralogous locations within the *HOX* clusters A, B, and C in mammals. This microRNA exhibits significant evolutionary conservation and has great complementarity to the transcripts of* HOXB8*, *HOXC8*, and *HOXD8*. Moreover, human *HOXB8*, *HOXC8*, *HOXD8*, and *HOXA7* were predicted to be targets of miR-196, followed by experimental validation 41.

Our findings have important clinical relevance. The identification of genetic variants associated with Wilms tumor susceptibility, such as the miR-196a2 rs11614913 C allele, facilitates the stratifying of children based on their genetic risk. Incorporating genetic testing for the rs11614913 C allele into routine screening panels for at-risk populations could improve the identification of high-risk individuals, guiding surveillance strategies, allowing for more targeted monitoring of those at greatest risk and potentially leading to earlier detection of Wilms tumor. Early identification of at-risk individuals through genetic markers can facilitate closer clinical follow-up and timely diagnostic evaluations, such as ultrasound screening for kidney abnormalities. Detecting Wilms tumor at an earlier stage significantly improves the prognosis and survival outcomes for affected children. In clinical practice, the presence of the miR-196a2 rs11614913 C allele could be used as part of a genetic risk assessment tool, prompting early imaging or other diagnostic measures in children with known genetic susceptibility. This approach aligns with precision medicine, where early intervention is tailored based on individual genetic profiles.

The limitations of this study were as follows. First, due to the rarity of Wilms tumor, the sample size of the case-control study was relatively moderate. Second, we investigated only one SNP in the study, and more functional SNPs in the *hsa-mir-196a* gene should be studied to explore the effect of gene-gene interactions. Third, to establish the role of the rs11614913 in the etiology of Wilms tumor, tumor tissue sample should be collected to determine the linkage between mature hsa-mir-196a expression and the rs11614913 genotypes (TT, TC, and CC). Fourth, the functional implications should be confirmed in Wilm's tumor cell lines. Fifth, environmental exposures, such as prenatal exposures, parental smoking, or other environmental influences, could also confound genetic associations. Due to the retrospective nature of our study and limitations in exposure data collection, these factors were unavailable. Finally, multiple statistical tests could increase the risk of type I errors, leading to false-positive findings. Replication in independent cohorts is a powerful method to strengthen the validity of the results and reduce concerns about false positives due to multiple comparisons. Therefore, our findings warrant validation in larger, well-designed case-control studies involving different ethnicities.

In conclusion, we demonstrated that the rs11614913 T>C is a moderate penetrant SNP associated with enhanced Wilms tumor predisposition in Chinese children. This risk genetic variant may have the potential to contribute to the Wilms tumor screening in the future.

## Supplementary Material

Supplementary table.

## Figures and Tables

**Table 1:** Association of *miR-196a2* rs11614913 T>C polymorphism with Wilms tumor susceptibility

Genotype	Cases (N=415)	Controls (N=936)	*P* ^ a^	Crude OR (95% CI)	*P*	Adjusted OR (95% CI) ^b^	*P* ^ b^
rs11614913 (HWE=0.351)							
TT	98 (23.61)	273 (29.17)		1.00		1.00	
TC	212 (51.08)	478 (51.07)		1.24 (0.93-1.64)	0.141	1.24 (0.93-1.64)	0.139
CC	105 (25.30)	185 (19.76)		**1.58 (1.13-2.21)**	**0.007**	**1.58 (1.14-2.21)**	**0.007**
Additive			0.007	**1.26 (1.06-1.49)**	**0.007**	**1.26 (1.06-1.49)**	**0.007**
Dominant	317 (76.39)	663 (70.83)	0.035	**1.33 (1.02-1.74)**	**0.035**	**1.33 (1.02-1.74)**	**0.034**
TT/TC	310 (74.70)	751 (80.24)		1.00		1.00	
CC	105 (25.30)	185 (19.76)	0.022	**1.38 (1.05-1.81)**	**0.023**	**1.38 (1.05-1.81)**	**0.023**

OR, odds ratio; CI, confidence interval. ^a^ χ^2^ test for genotype distributions between Wilms tumor patients and controls. ^b^ Adjusted for age and gender.

**Table 2:** Stratification analysis for the association of *miR-196a2* rs11614913 T>C polymorphism with Wilms tumor susceptibility

Variables	rs11614913 (cases/controls)		Crude OR	*P*	Adjusted OR ^a^	*P* ^a^
	TT	TC/CC	(95% CI)		(95% CI)	
Age, month						
≤18	37/116	105/288	1.14 (0.74-1.76)	0.545	1.15 (0.74-1.77)	0.538
>18	61/157	212/375	**1.46 (1.04-2.05)**	**0.031**	**1.45 (1.03-2.04)**	**0.032**
Gender						
Female	45/122	139/283	1.33 (0.90-1.98)	0.158	1.33 (0.89-1.98)	0.162
Male	53/151	178/380	1.34 (0.93-1.91)	0.116	1.34 (0.94-1.93)	0.108
Clinical stage						
I+II	68/273	201/663	1.22 (0.89-1.66)	0.213	1.21 (0.89-1.65)	0.226
III+IV	21/273	94/663	**1.84 (1.13-3.02)**	**0.015**	**1.92 (1.17-3.16)**	**0.010**

^a^ Adjusted for age and gender, omitting the corresponding stratify factor. OR, odds ratio; CI, confidence interval.
