# Supplementary material for: MiR-196a2 rs11614913 C Allele is Associated with Increased Wilms Tumor Susceptibility in Chinese Children
Source: J Cancer. 2025 Jan 1;16(2):479–85. doi: 10.7150/jca.102801 (PMC11685685; doi:10.7150/jca.102801)
Supplement: Supplementary file 1 — Supplementary table. [file jcav16p0479s1.pdf]

Table S1. Frequency distribution of selected variables in Wilms tumor patients and controls from Eastern China

Variables	Cases (n=416)		Controls (n=936)		P
	No.	%	No.	%	
Age range, month	0.33-151.33		0.001-156.00		0.898 [a]
Mean ± SD	34.09 ± 26.35		33.87 ± 30.88		
≤18	142	34.13	404	43.16	
>18	274	65.87	532	56.84	
Gender					0.742 [b]
Female	184	44.23	405	43.27	
Male	232	55.77	531	56.73	
Clinical stage					
I	124	29.81	/	/	
II	145	34.86	/	/	
III	77	18.51	/	/	
IV	38	9.13	/	/	
NA	32	7.69	/	/	

SD, standard deviation; NA, not available.

[a] T-test for age distribution between Wilms tumor patients and cancer-free controls.

[b] Two-sided χ^2 test for distributions between Wilms tumor patients and cancer-free controls.
